# Extraction of compression indices from maternal-fetal heart rate simultaneous signals

**DOI:** 10.1371/journal.pone.0313709

**Published:** 2025-01-02

**Authors:** Mariana S. Ramos, Susana Brás, Paula Pinto, Luísa Castro

**Affiliations:** 1 Department of Physics, University of Aveiro, Aveiro, Portugal; 2 IEETA, DETI, LASI, University of Aveiro, Aveiro, Portugal; 3 Hospital Particular Madeira, University of Madeira, Portugal; 4 MEDCIDS - Department of Community Medicine, Information and Decision in Health, Faculty of Medicine, University of Porto, Porto, Portugal; 5 CINTESIS@RISE - Center for Health Technology and Services Research, Faculty of Medicine, University of Porto, Porto, Portugal; Polytechnic University of Marche: Universita Politecnica delle Marche, ITALY

## Abstract

Intrapartum asphyxia is responsible for approximately 900 000 deaths per year worldwide. These numbers show the urgency of investing in the quality of fetal health care. The heart rate signal is a complex signal and sometimes behaves unpredictably. Thus, it becomes relevant to study approaches that take into account their complexity, namely non-linear compression-based methods. In this work, feature extraction was based on two approaches: univariate and bivariate. The univariate approach is concerned with the extraction of fetal, maternal and maternal-fetal compression ratios and the bivariate approach aims to extract compression indices from maternal-fetal heart rate simultaneous signals and of each of the signals individually over time. To understand how the features calculated in this work can be useful in distinguishing acidemic and non-acidemic cases, a classifier was applied. Three different classifiers were tested, and the one that proved to be more effective was the Support-Vector Machine. Furthermore, it was also possible to conclude that the input set of variables that provides a better performance (f1-score = 0.793) of the classifier is composed of the variables of maternal-fetal compression ratio, maternal-fetal normalized relative compression and maternal-fetal normalized compression distance, obtained through trend and residual signal, which indicates that slow and fast fluctuations on the heart rate time series are important in acidemia assessment.

## Introduction

Approximately 900 000 fetuses die as a result of intrapartum hypoxia each year and it is associated with another 1.1 million stillbirths during childbirth [1]. Intrapartum fetal hypoxia is characterized by a deficit of *O*_2_ caused by a pathological change in the components of the placenta. This situation leads to an accumulation of *CO*_2_, causing fetal acidemia and subsequently a lower pH in the fetal blood vessels [2]. Early detection of babies at risk of fetal acidemia may decrease the chance of post-diagnosis of cerebral palsy, neonatal encephalopathy or even death [2]. In this sense, adequate obstetric intervention depends on early diagnosis, which may be crucial in preventing fetal damage [3].

In the event of fetal metabolic acidosis, the umbilical artery pH is below 7.00 and the base deficit in the extracellular fluid is above 12 mmol/l [4–6]. However, there are already reports of undesired outcomes when the pH is below 7.05 and the base deficit in the extracellular fluid is above 10 mmol/l [5]. According to [5], in situations of acidosis, moderate to severe situations are considered when the pH is below 7.15. Fetal heart rate (FHR) is often used as an indicator of fetal well-being and its variation reflects the influence of the fetal autonomic nervous system and its sympathetic and parasympathetic components [4]. According to [7], health can be interpreted in three domains—simple, complex and chaotic. Specifically, FHR falls between the complex and chaotic domains, reflecting the low agreement and certainty among experts [7]. To mitigate this disagreement, auxiliary automatic systems have been developed over the last few years, based on linear characteristics, which have the potential to alert to possible pathological cases, providing specialists with an objective and automatic fetal health monitoring [8]. The fact that the fetal heart rate signal is complex and sometimes behaves unpredictably, makes it relevant to study other approaches that may be more appropriate, namely non-linear compression-based methods.

Regarding maternal-fetal heart rate simultaneous analysis, Khandoker et al. evaluated the direction and strength of maternal-fetal heart rate coupling in healthy and pathological cases [9]. Regarding the direction, they concluded that in pathological cases, compared to healthy cases, there is a lower influence of FHR on MHR and a greater influence of MHR on FHR. In the case of the coupling force, it must be evaluated taking into account the two directions mentioned above. Therefore, when it comes to the influence of FHR on MHR, the coupling force is much greater in pathological cases than in normal ones. On the other hand, in the influence of MHR on FHR, the coupling force for healthy cases is lower than for pathological cases [9]. In this sense, the interpretation of the two signals simultaneously seems to provide additional information in the detection of pathological cases when compared to the FHR analysis alone.

The main aim of this study was to access which compression indices, applied to FHR and MHR, perform better in the discrimination of acidemic from non-acidemic fetuses in the intrapartum period. For this aim, a real unicenter dataset of FHR and MHR simultaneous intrapartum signals was explored, and bivariate as well as univariate compression indices were computed.

Considering the personal and social impact of fetal pathologies, the Omniview SisPorto^®^ was developed to monitor and analyse the needed data for fetuses evaluation [10, 11]. This is based on guidelines provided by the International Federation of Gynaecology and Obstetrics (FIGO) for fetal monitoring and incorporates linear features such as baseline estimation, identification of accelerations and decelerations, and assessment of long- and short-term variability. In addition, this system provides real-time visual and audible alerts with different color codes, regarding fetal health state [8, 12].

Non-linear analysis of biological time series offers new possibilities to improve computer-aided diagnostic systems [13]. Gonçalves et al. calculated several linear and non-linear indices, including the average of the FHR, the very low, low, and high spectral frequencies, and entropies (Approximate—ApEn—and Sample Entropy—SampEn), and concluded that labor progression was associated with a significant increase in the domain of linear frequency indices, while non-linear indices decreased significantly [14].

A study carried out by Spilka et al. analyzed 217 signals of FHR where 94 of these signals corresponded to fetuses with acidemia. The team showed that adding non-linear methods to conventional features provided better accuracy in classifying healthy vs pathological fetuses. Sensitivity values of 73.4%, specificity of 76.3% and F-measure of 71.9% were obtained using linear and non-linear methods [15].

In 2013, Henriques et al. reported that through entropy and compression approaches it is possible to quantify different complexities of a system. To distinguish between hypoxic and healthy fetuses, the complexity in the initial and final segments of the FHR signal during the last hour of labor was calculated, considering segments of 5 and 10 minutes. It was concluded that both entropies and compressors allow distinguishing the two groups and that fetuses with lower umbilical artery blood pH have significantly lower entropy and compression indices, more markedly in the final segments [16].

Costa et al. studied how complexity varies between two groups of fetuses—acidemic and non-acidemic—using multiscale entropy indices. In this study, they showed that the complexity of FHR signals in the last two hours of labor was significantly higher in non-acidemic compared to acidemic fetuses and that, with the removal of the last 30 minutes before delivery from the analysis, the complexity remained lower for the acidemic group. These results support the hypothesis that an altered, i.e., less complex, temporal structure of FHR baseline fluctuations on multiple time scales may be a marker of acidemia [17].

More recently, Marques et al. analyzed the non-linear complexity in two databases, intrapartum and antepartum, with previously identified normal and pathological groups, through the calculation of ApEn and SampEn. When the whole examination is considered, the results obtained showed low entropy values with no evident difference between the non-pathological and pathological groups. Since the analysis did not reveal the intended result, the authors decided to process the time series in a 5-minute window long, computing a parameter per window. Changes were detected during specific long-term events, which allows us to infer that entropy can be considered as a first-level indicator for accelerations, decelerations and also for other physiological behaviors, such as sinusoidal FHR [13].

In 2016, Gonçalves et al. showed the potential of bivariate analysis and carried out an exploratory study to investigate how maternal heart rate and fetal heart rate variability changed during childbirth and how well they could detect newborn acidemia. Throughout this study, linear and non-linear indices for FHR and MHR were calculated separately and simultaneously, in a database of 51 pregnancies, where each signal was registered for 2 hours during labor (the same database used in this work). There was a significant increase in most linear indices of FHR and MHR and a decrease in entropy indices with labor progress. FHR alone and in conjunction with MHR (FHR-MHR) demonstrated the highest auROC values for fetal acidemia prediction, with 0.76 and 0.88 for umbilical arterial blood (UAB) pH thresholds of 7.20 and 7.15, respectively. The inclusion of the MHR in the bivariate analyses allowed obtaining a sensitivity and specificity of approximately 100 and 89.1%, respectively [11]. Moreover, there are other studies with different purposes that encourage analysis of the coupling of maternal-fetal heart rate [18–20].

Non-linear indices are not completely explored in the intrapartum fetal asphyxia diagnose. So, new studies should be performed to explore their potential as the one presented on this work.

## Materials

### Instruments

The system used to collect the data is called STAN^®^ 31 fetal monitor (NeoventaMedical, Gothemburg, Sweden). There are many STAN configurations, and the one used to collect these signals has several elements, namely, two sockets for heart rate acquisition, an electrocardiography sensor connected to three electrodes on the mother’s chest and finally, an ultrasound sensor placed on the mother’s abdomen. Usually, this is connected to Omniview SisPorto^®^, an intrapartum monitoring system, which is based on the linear characteristics of the heart rate signal to assist in the diagnosis of possible pathologies, through the generation of alerts [10, 11].

### Participants

It is important to point out that the signals analyzed during this work had been collected previously, within the scope of another project [10, 11, 21]. The data used throughout this work are completely anonymized. The collection was carried out following the principles of the Declaration of Helsinki, it was approved by the local ethics committee and all women gave their consent to participate [10].

The database contains heart rate biosignals, in which 61 women participated. Table 1 presents the maternal summarized characteristics of the study sample, namely their age, height, weight, systolic and diastolic blood pressure and gestational age. An analysis of the maternal characteristics revealed that all variables follow a normal distribution, with the exception of gestational age.

**Table 1:** Mean, standard deviation, median, interquartile range, maximum and minimum for maternal characteristics: Age (years), height (in centimeters), weight (in kilograms), systolic (S) and diastolic (D) blood pressure (in millimetre of mercury) and gestational age (weeks).

	Mean ± Standard Deviation	Median	Interquartile range	Maximum	Minimum
Age (years)	27.7 ± 5.4	28.0	7.0	38.0	16.0
Height (cm)	161.1 ± 5.3	162.0	6.0	173.0	150.0
Weight (kg)	71.4 ± 8.9	71.0	14.0	89.0	53.0
Blood pressure S (mmHg)	121.5 ± 11.0	121.0	18.0	146.0	100.0
Blood pressure D (mmHg)	73.5 ± 8.4	73.0	13.0	89.0	56.0
Gestational age (weeks)	39.7 ± 1.1	39.7	1.3	41.3	37.0

In this study, 34 male and 27 female fetuses were involved. In Table 2, it is possible to obtain information about the birthweight of the fetuses and the arterial pH of the umbilical arterial blood at birth, as well as the Apgar score at the 1st and 5th minutes. For more detail on data description, please refer to [10, 11, 21]. It is important to note that the weight and pH of the fetus exhibit a normal distribution, whereas Apgar scores at 1 and 5 minutes deviate from normality.

**Table 2:** Mean, standard deviation, median, interquartile range, maximum and minimum for fetal characteristics: Birthweight (in grams), umbilical arterial blood pH and Apgar scores at 1st and 5th minutes.

	Mean ± Standard Deviation	Median	Interquartile range	Maximum	Minimum
Birthweight (g)	3231.1 ± 331.2	3190.0	370.0	4045.0	2400.0
pH	7.24 ± 0.07	7.25	0.10	7.37	7.05
Apgar at 1st min	9.2 ± 0.6	9.0	1.0	10.0	9.0
Apgar at 5th min	9.9 ± 0.3	10.0	0.0	10.0	9.0

### Data

This database contains 2 signals: fetal heart rate collected through cardiotocography and maternal heart rate collected through electrocardiography. The signals were collected during two hours before childbirth. The data collection finished 10 or 30 minutes before the childbirth, in the case of vaginal or cesarean delivery, respectively [10, 21]. To facilitate the study, the signals were divided into a first hour, symbolized by h1, and a second hour, represented by h2, that is, the hour immediately before delivery. These signals were obtained with a sampling frequency of 4 Hz.

Since the database under study does not contain severe acidemic cases and similar to what was done in other studies [21, 22], it was decided that cases with a pH value lower than 7.15 will be considered for the acidemic group. Consequently, values above 7.15 were labeled non-acidemic. Based on the chosen criteria, there were 7 acidemic and 54 non-acidemic cases, making a total of 61 cases.

The study of the signals revealed that not all cases completed the two hours of data collection, obtaining a median of 119.27 and an interquartile distance of 1.08 minutes.

## Methods

### Pre-processing

The values of the extracted features are highly dependent on the quality of the signal pre-processing [15, 23]. The fetal signals had already been subjected to pre-processing, in order to reduce noise and artifacts. Briefly, this algorithm detects fetal heart rate beats below 60 bpm, above 200 bpm and beat-to-beat differences above 25 bpm. Values where this occurs are eliminated and replaced using interpolation, when the loss periods do not exceed 2 seconds. In case these conditions hold for longer periods, the previous segment of equal length and without loss is replicated [14]. For the maternal signal, a scale conversion obtained by adding 50 beats per minute to the original MHR values was performed and, after that, the signals were subjected to exactly the same algorithm, for more details refer to [10].

Therefore, the quality of the signals in the database was evaluated to determine possible losses and amount of information for each of the signals. The loss of each signal was calculated based on the number of missing samples compared to the total number of samples. The calculated loss for the FHR and MHR is an average of all the losses calculated for the fetal signal and the maternal signal, respectively. It was verified that there are no losses in the fetal signal, however, losses in the maternal signal were observed with an average percentage loss of 0.01.

Since the fetal signal does not present losses, the losses of the maternal-fetal combination correspond to the individual losses of the maternal signal. It was decided that the duration of the signal to be used would be that of the signal with a shorter duration, in order to standardize the size of the simultaneous signal segments to be used. This was only found in two cases in the database, in which the loss was not significant.

Since all the collected data may be useful, the FHR and the MHR were decomposed in their trend and residuals to be separately analyzed and processed.

The trend was estimated in order to obtain a smoothed signal. With this procedure it is possible to obtain the low frequency component, without resorting to frequency filters [24]. Additionally, through the residual, calculated by the difference between the original signal and the trend, the high frequency variations can be obtained. The trend can be calculated through the centered moving average (${\hat{m}}_{t}$), described by Eq 1. This type of smoothing procedure generally uses samples before and after the time when the smoothed estimate is to be calculated.

$$\begin{array}{c} {\hat{m}}_{t}=\frac{x_{t-\frac{w}{2}}+\cdots +x_{t-2}+x_{t-1}+x_{t}+x_{t+1}+x_{t+2}+\cdots +x_{t+\frac{w}{2}}}{w+1} \end{array}$$ (1)

where *t* is the index of the sample under study and *w* + 1 is the size of the window. The application of this type of method implies the loss of some points at the beginning and at the end of the signal, depending on the choice of the window.

Different sizes were defined for the window, more specifically 9, 17 and 25 samples, and their respective plots were visualized and analyzed. It was determined that the value that best preserves the signal, without softening it too much, that is, without removing possible pathophysiological information, would be a window of 17 samples. This window takes into account the 8 samples immediately preceding and following of the sample to be smoothed. The choice of the window size to use took into account its real-time implication, since the trend calculation is based on previous and following samples, which would cause a real-time delay. Therefore, considering a window of 17 samples, that is, 8 samples before and 8 samples after, only a delay of 2 seconds is generated, given that the signal sampling rate is 4 Hz.

Trend calculation as a pre-processing method was applied to all fetal and maternal heart rate signals. Thus, it was possible to obtain a signal with fewer variations.

As in the literature found on similar studies, the fetal and maternal heart rate signal segments were divided into 10-minute segments [14, 16, 17]. It is important to note that not all data contained the complete 2 hours, which implies that some segments do not reach the intended 10 minutes. These cases were analyzed in detail and only segments with more than 5 minutes of data were considered for the study, with 8 segments being discarded in total.

### Processing

In the scope of this study, it is intended to evaluate the non-linear relation between maternal and fetal data, which may provide useful information to study acidemic fetuses. Also, the maternal-fetal relation should be inspected, as, according to recent studies, it seems that there is a coupling between the signals in the presence of pathology. So, under this study, bivariate measures of compression are studied. The use of the methods configures a breakthrough line of research under the acidemia study. Considering that the FHR and MHR are signals that present variation that cannot be predicted, the evaluation of complexity and its potential alterations through time may conduce to new biomarkers of fetal acidemia. The complexity of the simultaneous FHR and MHR signals can be evaluated through some metrics, and throughout this study emphasis was given to the normalized relative compression (NRC) and to the normalized compression distance (NCD). Several studies have been developed using these metrics in various types of signals, especially in the area of biometric identification, which showed their potential [25–28].

### Data balacing

The database has a total of 54 non-acidemic cases and 7 acidemic cases, the fact that the database is unbalanced can influence the results obtained, namely when studying the behavior of groups. The number of existing cases in each group can be a highly relevant characteristic for the conclusions that can be drawn. For this reason, it was decided to proceed with its balancing.

The SMOTE data augmentation technique was used. SMOTE is an over-sampling approach in which the minority class is over-sampled by creating “synthetic” examples. Taking each example from the minority class and introducing symbolic examples along the line that connects any/all of the minority class’s closest neighbours, artificial samples are created as follows:

**1**. Calculate the difference between the feature vector in question and its nearest neighbor;

**2**. Multiply this difference by a random number between 0 and 1, and add it to the feature vector under consideration;

As a result, a random point along the line segment between two distinct features is chosen [29].

The SMOTE technique allowed to increase the acidemic dataset to 49 samples. This number was chosen to balance the two sets. Thus, the expanded database has a total of 103 cases, of which 49 are acidemic and 54 non-acidemic. It is important to mention that data augmentation is a technique commonly used in physiological data, given that pathological cases appear mostly in a smaller number of cases. Specifically, the SMOTE technique was chosen because it is widely used in the health area, particularly in these studies [30–32].

### Zlib

One of the approaches that may quantify the complexity of a signal is the compression. Compression is usually associated to file data reduction, but the mathematical principle behind it is much more complex. A compressor is able to estimate the data quantity, by the observation of patterns and repetition, and the ability to reduce information. So, if a compressor is able to severely reduce the size of a file, it indicates that the file has a lot of redundancy, and therefore there is a small amount of information in the file. By the opposite, if a compressor is not able to reduce the size of a file, it indicates that there is a small amount of repetitions, conducing to a high quantity of information in the file. In an analogue hypothesis, the information of the maternal and fetal heart rate is evaluated in the same way.

Compressors can be distinguished into two major classes: lossless and lossy. In lossless compressors every bit of data after decompression is restored, i.e., it is possible to reconstruct the original file. On the other hand, when it comes to lossy compressors, this is not possible as the file is permanently reduced, eliminating certain information during the compression process [16, 33]. One of the examples of a lossless data compression algorithm is the software library *zlib*, created by [34]. This algorithm is used on various types of data and by description fits the requirements of the data used in this study. Furthermore, it provides good compression on a wide range of data while using little system resources [34]. Since the results obtained were as expected, *zlib* was the compressor used.

In the scope of this work, the first approach was based on the analysis of maternal and fetal compression ratios, with the aim of verifying which features contributed most to the distinction between the two groups in question. The compression ratio is calculated from the Eq 2, translating the number of times its original length has been reduced.

$$\begin{array}{c} \text{Compression}\;\text{Ratio}=\frac{\left|x\right|}{\left|x^{*}\right|} \end{array}$$ (2)

where |*x*| is the length of the original segment and |x*| is the length of the compressed segment. This calculation can also be carried out inversely, obtaining the percentage of the reduction performed.

### Normalized relative compression

Normalized Relative Compression (NRC) is based on the notion of relative compression methods. This method is capable of compressing an object using exclusively the information of another [26]. The NRC of a string *x*, based on *y*, is defined as:

$$\begin{array}{c} \text{NRC}\left(x||y\right)=\frac{C(x||y)}{|x|log_{2}\left|A\right|}, \end{array}$$ (3)

where |*x*| is the length of the string *x* and |*A*| is the size of the alphabet. The size of the alphabet corresponds to the number of letters that will be used to encode the string. *C*(*x*||*y*) represents the compression of *x* relatively to *y* [35, 36]. This measure provides information about the amount of data in *x* that cannot be described by *y*. The NRC value is expected to be lower when comparing two data segments from the same source than when comparing data from different sources [25], i.e. when in presence of similar data the NRC is expected be lower than when the compared data is not similar. In the scope of this work, based on this measure, it will be inspected the similarity between maternal vs maternal data in disjoint time periods, fetal vs fetal data in disjoint time periods, maternal vs fetal and fetal vs maternal in the same time period. With this evaluation, it is intended to inspect the relation between the segment studying their similarity and consequently their dependence and coupling.

In this work, extended alphabet finite-context models (xaFCM) will be implemented to calculate *C*(*x*||*y*). Finite-context models have been useful in very different pattern recognition tasks since they have the ability to create similarity/dissimilarity measures [27]. This model conforms to the Markov property as it estimates the probability of the next sequence of *d* > 0 symbols from the information source using the *k* > 0 symbols immediately preceding it [36]. These parameters, called depth (*d*) and order context (*k*), must be adjusted depending on the type of data being processed, in order to improve compression performance.

It is important to point out that the calculation of this measure requires a symbolic representation of the signals, so it is necessary to quantize the signal. A quantization method aims to convert real continuous signals into symbolic ones. Quantization methods make use of the statistical characteristics of the signals allowing their representation in a symbolic space. The application of these methods presupposes loss of information, however it is minimized by the correct choice of the method to use.

So, the application of this compression method requires the choice of some factors. Among them, the choice of the quantizer that best preserves the information contained in the signals, the size of the alphabet and the parameters *d* and *k* stand out.

### Normalized compression distance

Normalized Compression Distance (NCD) consists of the distance between two signals [37]. The NCD can be obtained using Eq 4, as follows:

$$\begin{array}{c} NCD\left(x,y\right)=\frac{C\left(xy\right)-min\{C(x),C(y)\}}{max\{C(x),C(y)\}} \end{array}$$ (4)

Briefly, the signals are compressed with a certain fixed compressor and the bit size of the compressed version of a file *x* is recorded as *C*(*x*) [38]. Each pair of signals FHR and MHR, that is, *x* and *y*, are added in a single file *xy* and are compressed, generating a bit file *C*(*xy*). Subsequently, the difference between the length of the compressed file and the minimum lengths *C*(*x*), *C*(*y*) of the compressed versions of the two signals is calculated. Finally, we divided this difference by the maximum length of the compressed versions of the two signals, *C*(*x*), *C*(*y*), in order to normalize the values between 0 and 1, so that the relative comparison between instances is possible [38].

The *zlib* compressor was chosen and the fetal and maternal signals were compressed. From the values obtained for these compressions, it was possible to calculate the NCD.

### Feature extraction

Considering the goal of understanding the bivariate relation between maternal and fetal data in the prediction and evaluation of acidemia, in this work, the explored features were based on 3 different metrics—Compression Ratio, NRC and NCD.

Firstly, maternal, fetal and maternal-fetal compression ratios were extracted. These were calculated through expression 2, using the *zlib* compressor.

Secondly, in order to apply the NRC, some important factors were chosen, namely, the quantizer that best preserves the information contained in the signals, the size of the alphabet and the parameters *d* and *k*. After carrying out several tests it was concluded that the parameters that optimize the performance of the NRC are the Lloyds Max quantizer, an alphabet size of 20 letters and the parameters *d* = 6 and *k* = 6.

The calculation of the NRC was performed based on Eq 3. As explained earlier, this measure provides information about the amount of data in x that cannot be described by *y*. Compression was performed using simultaneous maternal and fetal heart rate signal segments. NRC is not symmetrical, so depending on the direction of the calculus, a different result is obtained. Thus, the NRC value can be calculated in two ways, i.e., calculating it based on the fetal signal or based on the maternal signal. When the calculation is based on the fetal signal, we are inspecting the dependency of the mother to the fetuses, by the opposite when the NRC is calculated based on the maternal signal, we are evaluating the fetal dependency to the mother. For each 10-minute segment, the NRC value was calculated in both ways, using the simultaneous fetal and maternal signal.

After calculating the measure in both ways, it was found that there was a higher occurrence of overfitting, i.e., values very close to 1, when calculating the NRC based on the fetal signal. Therefore, it was decided to use the NRC of the fetal signal given the sequence of the maternal signal. Thus, throughout this work, we will assume *x* as the fetal signal and *y* as the maternal signal, which empirically indicates the dependence of the fetuses to the mother. It is supposed that the dependence to the mother, in healthy foetuses should diminish through time, when approaching the delivery time [9].

In the analysis under study, the NRC was calculated with simultaneous maternal and fetal signal segments. However, it was also studied over time, both for the mother and the fetus, i.e., the NRC was calculated over time, in relation to the initial 10 minutes of signal. The study of the NRC over time for the mother and the fetus has as main objective to evaluate the evolution of each one, analyzing the similarity or dissimilarity of the fetus to itself and the mother to herself over time. The NRC calculated over time through the FHR and MHR separately, will allow to describe the dependency through time of each series.

Finally, the NCD was calculated for the maternal-fetal signal and, similarly to what was done before, it was also calculated for the mother and the fetus over time, separately.

This work was conducted using Python programming language.

### Machine learning models

Machine learning models allow to characterize the relation between data, finding relations, correlations, and patterns not accessible by traditional methods [39].

Being the machine learning models able to describe high dimensional data spaces, and considering that in clinical practice it is important to be able to describe and interpret the data relation with the prediction, under the scope of this work a layer of feature engineering was introduced, allowing the study of feature importance in the decision making.

To try to distinguish acidemic from non-acidemic cases, three classifiers were tested: Random Forest (RF), XGBoost and Support-Vector Machine (SVM).

Each tree in a random forest depends on the values of a random vector that was sampled randomly and with the same distribution for all the trees in the forest [40]. This type of classifier was one of the chosen to distinguish the two groups, as it is straightforward to interpret and self-explanatory.

Extreme Gradient Boosting, known as XGboost is a scalable, distributed gradient-boosted decision tree machine learning library. It is the leading machine learning library for regression, classification, and ranking problems because it includes parallel tree boosting, which allows solving many data science problems quickly and accurately [41].

To finish, the main objective of the Support-Vector Machine (SVM) is to find an optimal separation hyperplane between classes, which correctly classifies the data points. The main advantage of this classifier is that it tries to find the decision boundary between classes without really worrying about the number of cases available for each of the classes [42].

Random Forest, XGBoost and Support Vector Machine was selected based on their distinctive characteristics and suitability for the data set. RF and XGBoost, both tree-based algorithms, are widely recognized by their ability to handle complex and nonlinear relationships in data. In addition, these methods are inherently robust with unbalanced and underrepresented data sets due to packing techniques (RF) and pulse (XGBoost) they employ, which reduces bias and variance. In contrast, SVM was chosen to test a different approach based on distance to a hyperplane, allowing us to assess whether the model structure influenced performance. This selection ensures a comprehensive assessment of different algorithmic paradigms and their impact on the model results. Grid search was performed to correctly identify the optimal hyperparameters for each model. Specifically, for the SVM model, the kernel used was Radial Basis Function (RBF), with class_weight=‘balanced’ to handle class imbalance and probability = True to enable probability estimation.

In order to try to distinguish the two groups, the original database was used first. However, with only 7 pathological cases, the model was not able to define a strategy for data evaluation. Based on this evidence, it was decided to use the augmented database, which contains the original data together with the data from the artificially created acidemic cases.

#### Feature selection

The study of the mother-fetus relation was accessed on 10 minutes intervals, the inspected variables were: the compression ratio, the NRC and the NCD. Once the relation is intended to be evaluated the simultaneous metrics were calculated, but also the individual ones, the idea is to find the impact of each one in the process of acidemia evaluation.

It is important to mention that, the metric values referring to the first 30 minutes were eliminated, due to the fact that not all participants completed the 2 hours of signal and this causes inconsistencies on data length.

In this feature selection step, we study collinearity. As collinearity implies redundancy in the data, correlated features were reduced to one feature, when the correlation was above 0.5.

#### Cross-validation

The strategy for train data selection impacts the performance of the classifier. To select the training and test set, several approaches were tested, including leave-one-out, k-fold, stratified k-fold (number of splits = 20) and a specific validation designed to be more appropriate for the type of study data.

Leave-one-out and k-fold cross-validation would be acceptable options, however, these methods do not guarantee the existence of both classes in both training and testing sets. Since this factor is essential, the stratified k-fold was chosen. In this method, the folds are created by keeping track of the sample proportions for each class, thus ensuring that both training and testing sets will have both groups. However, both were tested and the high-performance values obtained with the first 3 cross-validation hypotheses may be affected by the increase in the database, as we are training and testing the model with data that may be replicas of each other. To rule out this hypothesis, we decided to test the model with 7 acidemic cases, originating from only one original acidemic case, and with 7 non-acidemic cases chosen at random. Model training was performed with the remaining cases, then, we trained the model with 89 cases (acidemic and non-acidemic) and tested it with 7 acidemic and 7 non-acidemic cases. In this way, we are guaranteeing that the model has never been in contact with one of the original cases, nor with its replicas, increasing the reliability of the performance metrics obtained with the testing set.

**Fig 1:**
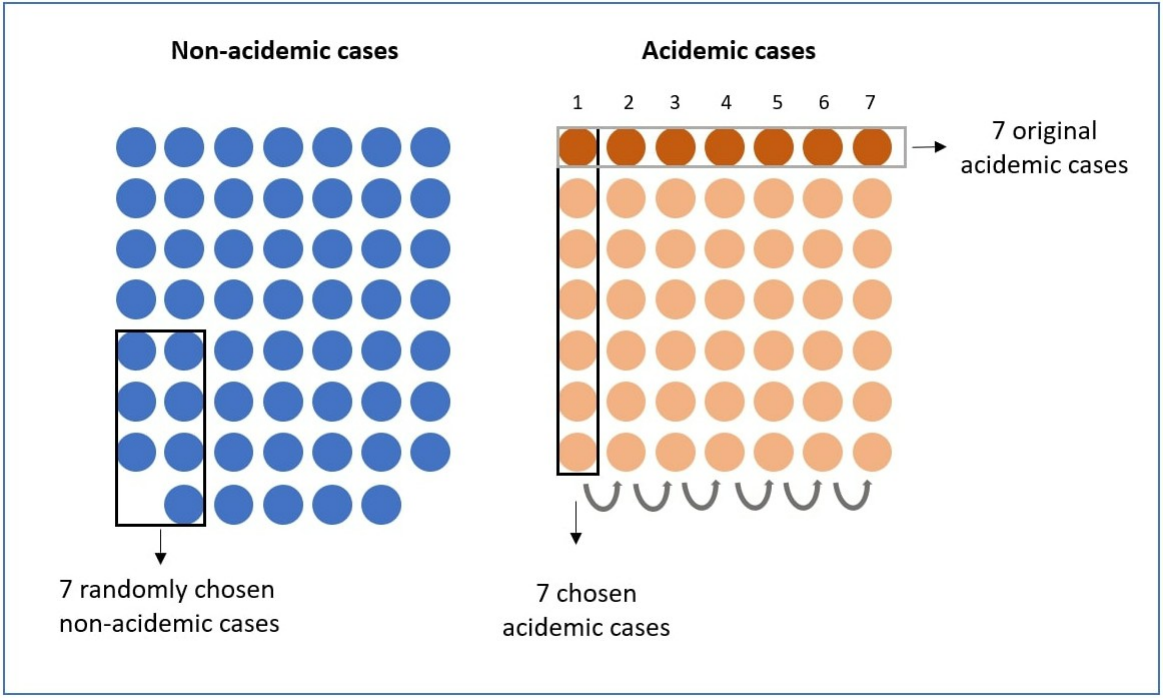
Choice of training and test set for validation. The test set is outlined by black rectangles, in the case of non-acidemic (in blue), 7 are chosen at random and in the case of acidemic (in orange), one of the columns numbered from 1 to 7 is chosen. The remaining cases that are not chosen for testing are used for training.

Fig 1 shows a schematic of the selection of the test set, outlined by the black rectangles, and the training set, i.e., the remaining cases. The original acidemic cases are outlined by a gray rectangle. Those immediately below are the acidemic cases obtained using data augmentation (SMOTE), remembering that each of the original acidemic cases generated 6 new ones. Since this validation is more appropriate to the type of data available for the study, the results shown hereafter are based on it.

## Results

The proposed approach was based on a high explainable concept, according to the needs in the understanding of the physiological process underlying the model. The process design, and the model implementation was carefully planned to accomplish an extra level of knowledge about the maternal-fetal relation, enabling an improvement in pathological identification.

The following characteristics will be used in the exploration of the classifiers: fetal compression ratio, maternal compression ratio, maternal-fetal compression ratio, fetal NRC, maternal NRC, maternal-fetal NRC, fetal NCD, maternal NCD and maternal-fetal NCD. Each of these characteristics has two calculated values in each 10-minute segment, for the trend and for the residual.

Nine variable sets were tested using the three classifiers. In the first eight sets, several classifiers were used, in which the input data were the features described below for the trend and for the residue, individually. Only the last test developed, test I, aggregates features from the trend and the residual at the same time as input data. The features used for each of the tests performed are described below:

- **Test A**: compression ratio, NRC and NCD for the trend of maternal, fetal and maternal-fetal signals;
- **Test B**: compression ratio, NRC and NCD for the residual of maternal, fetal and maternal-fetal signals;
- **Test C**: compression ratio, NRC and NCD for the trend of maternal and fetal signals;
- **Test D**: compression ratio, NRC and NCD for the residual of maternal and fetal signals;
- **Test E**: compression ratio, NRC and NCD for the trend of maternal-fetal signals;
- **Test F**: compression ratio, NRC and NCD for the residual of maternal-fetal signals;
- **Test G**: NRC and NCD for the trend of maternal-fetal signals;
- **Test H**: NRC and NCD for the residual of maternal-fetal signals;
- **Test I**: compression ratio, NRC and NCD for the trend and residual of maternal-fetal signals.

Test A contains all the features used in this paper. The remaining tests are variations of test A, in which different features are selected. In order to facilitate the study of the distribution of features, this study was carried out only for test A. Table 3 shows the mean and standard deviation for features that follow a normal distribution, and Table 4 shows the median and interquartile range for those that do not follow a normal distribution.

**Table 3:** Mean and standard deviation for all Test A features that follow a normal distribution.

Feature	Mean	Standard Deviation	Feature	Mean	Standard Deviation
maternal-fetal NCD—h2-c	0,87	0,03	fetal NRC—h2-b	0,55	0,18
maternal-fetal NRC—h2-a	0,59	0,16	fetal NRC—h2-f	0,55	0,17
maternal-fetal NRC—h2-c	0,61	0,18	fetal NCD—h2-f	0,86	0,02

**Table 4:** Median and interquartile range for all features in Test A that do not follow a normal distribution.

Feature	Median	Interquartile Range	Feature	Median	Interquartile Range
fetal compression ratio—h1-d	6,53	1,97	maternal-fetal NRC—h1-e	0,61	0,28
fetal compression ratio—h1-e	6,71	1,89	maternal-fetal NRC—h1-f	0,55	0,32
fetal compression ratio—h1-f	6,35	1,94	maternal-fetal NRC—h2-b	0,48	0,27
fetal compression ratio—h2-a	7,35	2,15	maternal-fetal NRC—h2-d	0,54	0,21
fetal compression ratio—h2-b	7,72	1,67	maternal-fetal NRC—h2-e	0,59	0,28
fetal compression ratio—h2-c	7,09	1,60	maternal-fetal NRC—h2-f	0,58	0,21
fetal compression ratio—h2-d	7,26	1,69	maternal NRC—h1-e	0,63	0,16
fetal compression ratio—h2-e	7,51	2,22	maternal NRC—h1-f	0,66	0,22
fetal compression ratio—h2-f	6,55	1,32	maternal NRC—h2-a	0,64	0,27
maternal compression ratio—h1-d	4,83	1,23	maternal NRC—h2-b	0,70	0,19
maternal compression ratio—h1-e	5,05	0,86	maternal NRC—h2-c	0,64	0,24
maternal compression ratio—h1-f	5,00	1,58	maternal NRC—h2-d	0,68	0,20
maternal compression ratio—h2-a	4,88	1,32	maternal NRC—h2-e	0,70	0,17
maternal compression ratio—h2-b	4,98	1,43	maternal NRC—h2-f	0,66	0,16
maternal compression ratio -h2-c	4,84	1,23	fetal NRC—h1-e	0,53	0,22
maternal compression ratio—h2-d	5,23	1,35	fetal NRC—h1-f	0,51	0,24
maternal compression ratio—h2-e	5,10	1,38	fetal NRC—h2-a	0,38	0,25
maternal compression ratio—h2-f	5,35	1,27	fetal NRC—h2-c	0,54	0,30
maternal-fetal compression ratio—h1-d	6,21	1,01	fetal NRC—h2-d	0,53	0,32
maternal-fetal compression ratio—h1-e	6,13	1,13	fetal NRC—h2-e	0,76	0,15
maternal-fetal compression ratio—h1-f	6,33	1,50	maternal NCD—h1-e	0,86	0,03
maternal-fetal compression ratio—h2-a	6,45	0,71	maternal NCD—h1-f	0,86	0,05
maternal-fetal compression ratio—h2-b	6,34	1,28	maternal NCD—h2-a	0,85	0,03
maternal-fetal compression ratio -h2-c	6,18	1,01	maternal NCD—h2- b	0,85	0,04
maternal-fetal compression ratio—h2-d	6,54	0,96	maternal NCD—h2-c	0,85	0,03
maternal-fetal compression ratio—h2-e	6,22	0,89	maternal NCD—h2- d	0,85	0,03
maternal-fetal compression ratio—h2-f	6,40	1,13	maternal NCD—h2-e	0,86	0,03
maternal-fetal NCD—h1-d	0,86	0,04	maternal NCD—h2-f	0,86	0,04
maternal-fetal NCD—h1-e	0,88	0,04	fetal NCD—h1-e	0,86	0,03
maternal-fetal NCD—h1-f	0,88	0,04	fetal NCD—h1-f	0,85	0,05
maternal-fetal NCD—h2-a	0,87	0,06	fetal NCD—h2-a	0,86	0,06
maternal-fetal NCD—h2-b	0,87	0,07	fetal NCD—h2-b	0,86	0,05
maternal-fetal NCD—h2-d	0,88	0,06	fetal NCD—h2-c	0,86	0,03
maternal-fetal NCD—h2-e	0,88	0,05	fetal NCD—h2-d	0,85	0,04
maternal-fetal NCD—h2-f	0,86	0,04	fetal NCD—h2-e	0,86	0,05
maternal-fetal NRC—h1-d	0,61	0,39			

The distribution of features was analyzed for all cases and also separately by classes. The results of the class-based tests are presented in Tables 5–7. While for non-acidemic cases some features follow a normal distribution and others do not, in acidemic cases no feature follows a normal distribution. This discrepancy can be explained by the fact that some acidemic cases were obtained through data augmentation, which may have reduced the diversity of the data.

**Table 5:** Mean and standard deviation for all Test A features corresponding to non-acidemic cases that follow a normal distribution.

Feature	Mean	Standard Deviation	Feature	Mean	Standard Deviation
maternal-fetal compression ratio—h2-f	6.84	0.78	maternal NRC—h2-e	0.68	0.13
maternal-fetal NCD—h1-d	0.88	0.03	maternal NRC—h2-f	0.67	0.15
maternal-fetal NCD—h1-e	0.88	0.04	fetal NRC—h1-e	0.56	0.23
maternal-fetal NCD—h1-f	0.87	0.04	fetal NRC—h1-f	0.55	0.24
maternal-fetal NCD—h2-a	0.88	0.04	fetal NRC—h2-b	0.54	0.20
maternal-fetal NCD -h2-b	0.88	0.03	fetal NRC—h2-c	0.54	0.19
maternal-fetal NCD—h2-c	0.88	0.03	fetal NRC—h2-d	0.56	0.20
maternal-fetal NCD—h2-d	0.87	0.03	fetal NRC—h2-e	0.67	0.18
maternal-fetal NCD—h2-e	0.88	0.03	fetal NRC—h2-f	0.53	0.19
maternal-fetal NCD—h2-f	0.86	0.03	maternal NCD—h1-e	0.86	0.03
maternal-fetal NRC—h1-d	0.60	0.19	maternal NCD—h1-f	0.86	0.03
maternal-fetal NRC—h1-e	0.62	0.20	maternal NCD—h2-a	0.86	0.03
maternal-fetal NRC—h1-f	0.57	0.19	maternal NCD—h2-b	0.86	0.03
maternal-fetal NRC—h2-a	0.61	0.20	maternal NCD—h2-c	0.86	0.03
maternal-fetal NRC—h2-b	0.61	0.19	maternal NCD—h2-d	0.86	0.03
maternal-fetal NRC—h2-c	0.61	0.20	maternal NCD—h2-e	0.87	0.03
maternal-fetal NRC—h2-d	0.57	0.19	maternal NCD—h2-f	0.87	0.03
maternal-fetal NRC—h2-e	0.58	0.18	fetal NCD—h1-e	0.86	0.04
maternal-fetal NRC—h2-f	0.62	0.16	fetal NCD—h1-f	0.85	0.04
maternal NRC—h1-e	0.69	0.14	fetal NCD—h2-b	0.86	0.03
maternal NRC—h1-f	0.66	0.15	fetal NCD—h2-c	0.86	0.04
maternal NRC—h2-a	0.70	0.16	fetal NCD—h2-d	0.86	0.03
maternal NRC—h2-b	0.69	0.15	fetal NCD—h2-e	0.86	0.03
maternal NRC—h2-c	0.66	0.15	fetal NCD—h2-f	0.86	0.03
maternal NRC—h2-d	0.65	0.14			

**Table 6:** Median and interquartile range for all Test A features corresponding to non-acidemic cases that do not follow a normal distribution.

Feature	Median	Interquartile Range	Feature	Median	Interquartile Range
fetal compression ratio—h1-d	6.79	2.29	maternal compression ratio—h2-d	5.04	1.11
fetal compression ratio—h1-e	6.86	1.76	maternal compression ratio—h2-e	5.28	1.22
fetal compression ratio—h1-f	7.02	2.84	maternal compression ratio—h2-f	5.72	1.50
fetal compression ratio—h2-a	7.10	1.88	maternal-fetal compression ratio—h1-d	6.06	1.01
fetal compression ratio—h2-b	7.04	1.53	maternal-fetal compression ratio—h1-e	6.14	1.04
fetal compression ratio—h2-c	7.09	2.06	maternal-fetal compression ratio—h1-f	6.46	1.33
fetal compression ratio—h2-d	7.40	1.85	maternal-fetal compression ratio—h2-a	6.33	1.11
fetal compression ratio—h2-e	7.15	1.97	maternal-fetal compression ratio—h2-b	6.18	1.14
fetal compression ratio—h2-f	6.66	1.13	maternal-fetal compression ratio—h2-c	6.25	1.41
maternal compression ratio—h1-d	4.79	0.96	maternal-fetal compression ratio—h2-d	6.55	0.92
maternal compression ratio—h1-e	4.85	0.97	maternal-fetal compression ratio—h2-e	6.69	0.83
maternal compression ratio—h1-f	4.99	1.34	fetal NRC—h2-a	0.43	0.24
maternal compression ratio—h2-a	5.12	1.39	fetal NCD—h2-a	0.87	0.05
maternal compression ratio—h2-b	4.83	1.41			

**Table 7:** Median and interquartile range for all Test A features corresponding to acidemic cases that do not follow a normal distribution.

Feature	Median	Interquartile Range	Feature	Median	Interquartile Range
fetal compression ratio—h1-d	5,96	1,97	maternal-fetal NRC—h2 -a	0,58	0,17
fetal compression ratio—h1-e	6,26	2,79	maternal-fetal NRC—h2-b	0,41	0,29
fetal compression ratio—h1-f	6,23	1,74	maternal-fetal NRC—h2-c	0,60	0,24
fetal compression ratio—h2-a	8,14	2,65	maternal-fetal NRC—h2- d	0,54	0,21
fetal compression ratio—h2-b	8,61	2,05	maternal-fetal NRC—h2-e	0,63	0,21
fetal compression ratio—h2-c	7,09	2,05	maternal-fetal NRC—h2-f	0,58	0,21
fetal compression ratio—h2-d	7,26	1,87	maternal NRC—h1-e	0,62	0,20
fetal compression ratio—h2-e	7,72	2,37	maternal NRC—h1-f	0,74	0,37
fetal compression ratio—h2-f	6,45	1,71	maternal NRC—h2 -a	0,60	0,33
maternal compression ratio—h1-d	4,98	1,87	maternal NRC—h2-b	0,70	0,19
maternal compression ratio—h1-e	5,27	1,18	maternal NRC—h2-c	0,58	0,31
maternal compression ratio—h1-f	5,00	2,25	maternal NRC—h2-d	0,69	0,20
maternal compression ratio—h2-a	4,68	1,32	maternal NRC—h2-e	0,74	0,17
maternal compression ratio—h2-b	5,15	1,45	maternal NRC—h2-f	0,66	0,16
maternal compression ratio—h2-c	4,84	1,54	fetal NRC—h1-e	0,53	0,22
maternal compression ratio—h2-d	5,47	1,69	fetal NRC—h1-f	0,48	0,21
maternal compression ratio—h2-e	4,86	0,98	fetal NRC—h2-a	0,36	0,22
maternal compression ratio—h2-f	4,66	0,94	fetal NRC—h2-b	0,60	0,22
maternal-fetal compression ratio—h1-d	6,21	1,02	fetal NRC-h2-c	0,54	0,37
maternal-fetal compression ratio—h1-e	6,13	2,12	fetal NRC—h2-d	0,53	0,38
maternal-fetal compression ratio—h1-f	5,80	2,29	fetal NRC—h2-e	0,77	0,14
maternal-fetal compression ratio—h2-a	6,45	0,61	fetal NRC—h2-f	0,57	0,17
maternal-fetal compression ratio—h2-b	6,66	1,67	maternal NCD—h1-e	0,85	0,06
maternal-fetal compression ratio—h2-c	6,18	1,01	maternal NCD—h1-f	0,86	0,07
maternal-fetal compression ratio—h2-d	6,54	1,43	maternal NCD—h2-a	0,84	0,03
maternal-fetal compression ratio—h2-e	5,91	0,45	maternal NCD—h2-b	0,84	0,03
maternal-fetal compression ratio—h2-f	6,03	0,84	maternal NCD—h2-c	0,84	0,03
maternal-fetal NCD—h1-d	0,86	0,01	maternal NCD—h2-d	0,85	0,02
maternal-fetal NCD—h1-e	0,88	0,04	maternal NCD—h2-e	0,84	0,04
maternal-fetal NCD—h1-f	0,88	0,05	maternal NCD- h2-f	0,86	0,02
maternal-fetal NCD—h2-a	0,86	0,06	fetal NCD—h1-e	0,86	0,03
maternal-fetal NCD—h2-b	0,87	0,07	fetal NCD—h1-f	0,85	0,07
maternal-fetal NCD—h2-c	0,87	0,06	fetal NCD—h2-a	0,85	0,06
maternal-fetal NCD—h2-d	0,89	0,07	fetal NCD—h2-b	0,88	0,08
maternal-fetal NCD—h2-e	0,89	0,07	fetal NCD-h2-c	0,86	0,03
maternal-fetal NCD—h2-f	0,88	0,06	fetal NCD—h2-d	0,85	0,03
maternal-fetal NRC—h1-d	0,67	0,49	fetal NCD—h2-e	0,85	0,06
maternal-fetal NRC—h1-e	0,69	0,28	fetal NCD—h2-f	0,86	0,04
maternal-fetal NRC—h1-f	0,55	0,39			

The performance values obtained in all tests performed, using the three classifiers under study—Random Forest, XGBoost and SVM—showed that the one that allows a better distinction between groups is the SVM, as can be seen in the Tables 8–10. For this reason, the other two classifiers were discarded and the analysis and discussion will focus on the SVM classifier. Table 10 shows the performance metrics obtained for each of the variable sets.

**Table 8:** Accuracy, recall, specificity, precision, f1-score, and AUC values were obtained for the Random Forest classifier for the different tests (A,B,C,D,E,F,G,H,I).

Test	A	B	C	D	E	F	G	H	I
Accuracy	0.541	0.459	0.531	0.449	0.582	0.449	0.469	0.520	0.520
Recall	0.143	0.000	0.143	0.000	0.286	0.000	0.000	0.143	0.143
Specificity	0.939	0.918	0.918	0.898	0.878	0.898	0.939	0.898	0.898
Precision	0.125	0.000	0.125	0.000	0.250	0.000	0.000	0.111	0.143
F1-score	0.133	0.000	0.133	0.000	0.267	0.000	0.000	0.125	0.143
AUC	0.541	0.459	0.531	0.449	0.582	0.449	0.469	0.520	0.520

**Table 9:** Accuracy, recall, specificity, precision, f1-score, and AUC values were obtained for the XGBoost classifier for the different tests (A,B,C,D,E,F,G,H,I).

Test	A	B	C	D	E	F	G	H	I
Accuracy	0.449	0.449	0.520	0.449	0.582	0.449	0.541	0.520	0.439
Recall	0.000	0.000	0.143	0.000	0.286	0.000	0.143	0.143	0.000
Specificity	0.898	0.898	0.898	0.898	0.878	0.898	0.939	0.898	0.878
Precision	0.000	0.000	0.111	0.000	0.250	0.000	0.125	0.111	0.000
F1-score	0.000	0.000	0.125	0.000	0.267	0.000	0.133	0.125	0.000
AUC	0.449	0.449	0.520	0.449	0.582	0.449	0.541	0.520	0.439

**Table 10:** Accuracy, recall, specificity, precision, f1-score, and AUC values were obtained for the Support-vector machine classifier for the different tests (A,B,C,D,E,F,G,H,I). In shaded gray are the tests that only used the residual signal as input data and in blue is the only test that has as input data features from the trend and the residual signals.

Test	A	B	C	D	E	F	G	H	I
Accuracy	0.531	0.704	0.439	0.694	0.694	0.418	0.490	0.429	0.735
Recall	0.571	0.571	0.449	0.571	0.857	0.286	0.143	0.000	1.000
Specificity	0.490	0.837	0.429	0.816	0.531	0.551	0.837	0.857	0.469
Precision	0.382	0.490	0.281	0.504	0.591	0.168	0.125	0.857	0.660
F1-score	0.458	0.526	0.338	0.535	0.699	0.211	0.133	0.000	0.793
AUC	0.531	0.704	0.439	0.694	0.694	0.418	0.490	0.429	0.735

The first two tests use all extracted features as input data, for the trend and for the residue, respectively. Through the results observed in Table 10, it can be concluded that the features from the residue (test B) have a superior ability to distinguish the two groups, in relation to the same features extracted through the trend (test A). The same was verified for tests C and D, in which the input data only contains maternal and fetal metrics, separately. These results may indicate a high discriminative power of the fast fluctuations of FHR and MHR time series, in comparison with the slow fluctuations.

To understand the impact of the maternal-fetal relationship, maternal-fetal features were given as input data in tests E and F. It is possible to observe, in Table 10, that the performance values are higher in the trend when compared to the tests A and C. On the other hand, the performance values related to the residue are lower (test F), in comparison with the two previous ones performed for the residue (tests B and D). Contrary to what was observed in the previous tests, in tests E and F, the trend signal has more discriminatory power than the same characteristics obtained from the residual signal, indicating that the slow variation is also an important part of the process.

To evaluate the effect of maternal-fetal compression ratio on classifier performance, the input data for tests G and H were the same as the two previous tests excluding the compression ratio. Comparing these tests with E and F, it is possible to conclude that the compression ratio plays an important role in distinguishing the two groups since the performance values have decreased. In the trend, the value of f1-score decreases from 0.699 to 0.133 and in the residual from 0.211 to 0.

In the last test, it was decided to combine the maternal-fetal features (compression ratio, NRC and NCD) of trend and residual in the same set of input. This was the set of variables that provided the best performance of the classifier (f1-score = 0.793), which indicates that slow and fast fluctuations in the time series of heart rate are important in the acidemia evaluation, as well as the simultaneous evaluation of the maternal-fetal relation.

Based on Table 10, it can be concluded that maternal-fetal features play a fundamental role in the performance of the classifier.

**Fig 2:**
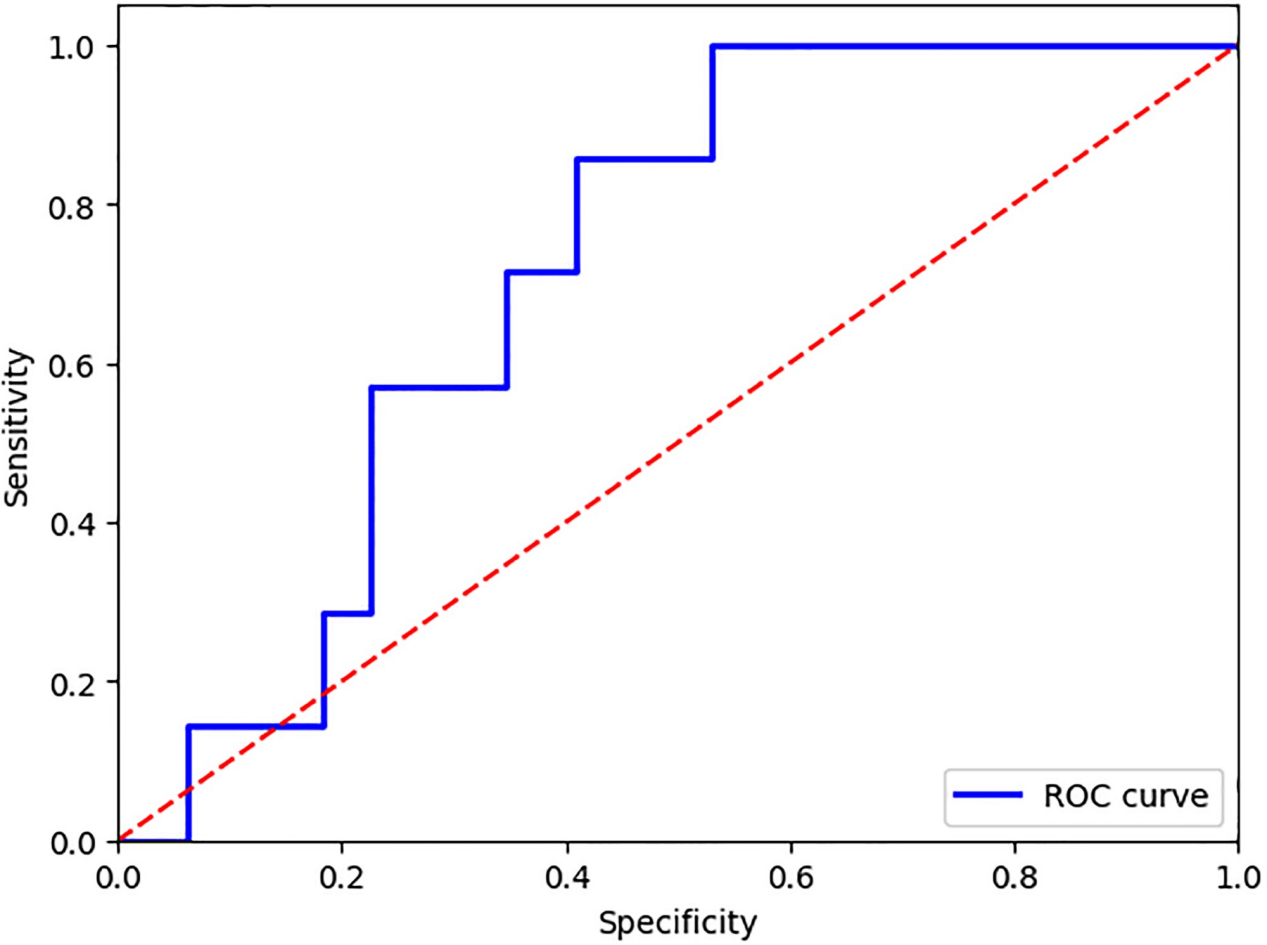
Representation of the ROC curve for Test I.

In the Fig 2, we present the ROC curve of test I. Analysis of the ROC curve reveals that the SVM model achieved a rate of true positives higher than the rate of false positives in most of the graph. The dashed red line represents the performance of a random classifier, while the blue curve, which positions itself above that line, suggests that the model is performing more accurate ratings than chance. In summary, the performance of the SVM model for the classification between Acidemic and Non-acidemic is moderate. Although the model shows an above random discrimination capacity, evidencing effectiveness in the distinction between the two classes, the presence of a significant rate of false positives at some points indicates the need for adjustments. Despite these limitations, the current performance of the model demonstrates a promising potential.

## Discussion

This study shows the potential of using features from the mother, together with those from the fetus, is shown to improve the detection of fetal acidemia, which corroborates the study presented earlier [10].

Furthermore, it is extremely interesting to evaluate the recall (or sensitivity) and the specificity values obtained. A system implemented in clinical practice is ideal when its specificity and sensitivity are 100% so that there is no margin of error when a result is presented. However, this is not always possible. Cardiocotography has been seen as one of the main causes of the increase in the cesarean rate [43], so although the main objective is to correctly detect fetuses with fetal acidemia, assessed by sensitivity, it is important to also control the specificity, not to waste resources. Test I has a value of 1 for recall and 0.469 for specificity, so we can infer that it has a greater probability of detecting an acidemic fetus, but its specificity, i.e., the probability of detecting a non-acidemic fetus is low, which in this particular application may imply an increase in cesarean rates. There are limited studies that incorporate both maternal and fetal signals. However, in 2016, Gonçalves et al. demonstrated the potential of bivariate analysis by exploring how maternal heart rate (MHR) and fetal heart rate (FHR) variability change during labor and predict fetal acidemia. By analyzing both signals together, the prediction of fetal acidemia improved, reaching a sensitivity of 100% and specificity of 89.1%, with a high predictive accuracy (auROC of 0.88 for pH < 7.15). Other studies, such as those by [3, 17, 44], have shown similar potential using only fetal signals.

## Conclusion

This study enabled to explain and interpret the physiological data that may indicate a support in decision making of acidemia evaluation. The use of maternal-fetal data introduces an extra information that allow to discriminate the condition with better feasibility, indicating that the maternal-fetal relation presents different patterns on pathological and non-pathological cases. Maternal-fetal compression ratio, maternal-fetal NRC and maternal-fetal NCD, obtained through trend and residual signals presented good results, with an f1-score value of 0.793.

Based on this, it is important to highlight:

1. the relevance of including features from the trend and the residue simultaneously, which indicates that slow and fast fluctuations of the heart rate time series are important in acidemia evaluation.
2. using compression indices, namely the compression ratio, the NRC and the NCD, show promising results in distinguishing acidemic from non-acidemic fetuses, reinforcing the relevance of non-linear metrics in the detection of fetal acidemia.
3. underline that the features that optimize the performance of the classifier are maternal-fetal. These show the potential of including features from the mother together with features from the fetus, as they help in decision making, allowing for better performances and thus improving fetal monitoring effectiveness.

### Limitations and future work

This study has several limitations that should be noted. The primary limitation is the small number of acidemic cases. While validating the results with a larger database might yield better outcomes, the low incidence of the disease could still pose a challenge [44]. Another significant limitation is the use of synthetic data resampling to address class imbalance. The pathological class had few representative cases, necessitating the use of the SMOTE technique to increase sampling, which could introduce biases into the model’s predictions [45]. Additionally, the study used a pH cutoff value of 7.15. Although some research adopts this threshold, other studies use lower pH values. Due to the absence of severe acidemia cases in the database, 7.15 was chosen as the cutoff.

A current limitation of this study is the lack of a standardized approach for selecting cutoff points. Future research should focus on validating these cutoffs with independent datasets and larger sample sizes to enhance their clinical applicability and enable meaningful comparisons across models.

Future research should primarily focus on addressing the issue of class imbalance, developing methods that ensure fair decision-making without biasing the majority class. Here are some suggested areas for future exploration:

Investigating the impact of varying time windows for segmenting signals (in this study, 10-minute non-overlapping intervals were used). Exploring the NRC calculation by adjusting the depth (d) and order context (k) values. Additionally, other studies have shown that non-linear measures, such as entropy, can effectively discriminate between acidemic and non-acidemic cases [13, 16]. Therefore, it may be beneficial to develop a classifier that combines multiple non-linear features, like compression and entropy, with the linear features currently used in clinical practice.

## References

[1] Lawn JE, Manandhar A, Haws RA, Darmstadt GL. Reducing one million child deaths from birth asphyxia—A survey of health systems gaps and priorities. Health Research Policy and Systems. 2007;5:1–10. doi: 10.1186/1478-4505-5-4 17506872 PMC1888686

[2] Georgoulas G, Stylios C, Groumpos P. Feature extraction and classification of fetal heart rate using wavelet analysis and support vector machines. International Journal on Artificial Intelligence Tools. 2006;15(03):411–432. doi: 10.1142/S0218213006002746

[3] Zarmehri MN, Castro L, Santos J, Bernardes J, Costa A, Santos CC. On the prediction of foetal acidaemia: A spectral analysis-based approach. Computers in Biology and Medicine. 2019;109(October 2018):235–241. doi: 10.1016/j.compbiomed.2019.04.041 31085380

[4] Garabedian C, De Jonckheere J, Butruille L, Deruelle P, Storme L, Houfflin-Debarge V. Understanding fetal physiology and second line monitoring during labor. Journal of Gynecology Obstetrics and Human Reproduction. 2017;46(2):113–117. doi: 10.1016/j.jogoh.2016.11.005 28403965

[5] Ayres-De-Campos D, Arulkumaran S. FIGO consensus guidelines on intrapartum fetal monitoring: Physiology of fetal oxygenation and the main goals of intrapartum fetal monitoring. International Journal of Gynecology and Obstetrics. 2015;131(1):5–8. doi: 10.1016/j.ijgo.2015.06.020 26433399

[6] The American College of Obstetricians and Gynecologists. Neonatal Encephalopathy and Neurologic Outcome. Postgraduate Obstetrics Gynecology. 2014;34(18):6. doi: 10.1097/01.PGO.0000453617.48477.32 24785633

[7] Plsek PE, Greenhalgh T. Complexity science: The challenge of complexity in health care. [Article 1 in series of 4]. BMJ (Clinical research ed). 2001;323(7313):625–8. doi: 10.1136/bmj.323.7313.625 11557716 PMC1121189

[8] Nunes I, Ayres-De-Campos D, Figueiredo C, Bernardes J. An overview of central fetal monitoring systems in labour. Journal of Perinatal Medicine. 2013;41(1):93–99. doi: 10.1515/jpm-2012-0067 23093259

[9] Khandoker AH, Schulz S, Al-Angari HM, Voss A, Kimura Y. Alterations in maternal-fetal heart rate coupling strength and directions in abnormal fetuses. Frontiers in Physiology. 2019;10(APR):1–12. doi: 10.3389/fphys.2019.00482 31105586 PMC6498890

[10] Pinto P, Bernardes J, Costa-Santos C, Amorim-Costa C, Silva M, Ayres-de Campos D. Development and evaluation of an algorithm for computer analysis of maternal heart rate during labor. Computers in Biology and Medicine. 2014;49(1):30–35. doi: 10.1016/j.compbiomed.2014.03.007 24727565

[11] Gonçalves H, Pinto P, Silva M, Ayres-de Campos D, Bernardes J. Toward the improvement in fetal monitoring during labor with the inclusion of maternal heart rate analysis. Medical and Biological Engineering and Computing. 2016;54(4):691–699. doi: 10.1007/s11517-015-1359-7 26219610

[12] Ayres-de Campos D, Rei M, Nunes I, Sousa P, Bernardes J. SisPorto 4.0–computer analysis following the 2015 FIGO Guidelines for intrapartum fetal monitoring. Journal of Maternal-Fetal and Neonatal Medicine. 2017;30(1):62–67. doi: 10.3109/14767058.2016.1161750 26940372

[13] Marques JAL, Cortez PC, Madeiro JPV, de Albuquerque VHC, Fong SJ, Schlindwein FS. Nonlinear characterization and complexity analysis of cardiotocographic examinations using entropy measures. Journal of Supercomputing. 2020;76(2):1305–1320. doi: 10.1007/s11227-018-2570-8

[14] Gonçalves H, Rocha AP, Ayres-de Campos D, Bernardes J. Linear and nonlinear fetal heart rate analysis of normal and acidemic fetuses in the minutes preceding delivery. Medical and Biological Engineering and Computing. 2006;44(10):847–855. doi: 10.1007/s11517-006-0105-6 16988896

[15] Spilka J, Chudáček V, Koucký M, Lhotská L, Huptych M, Janků P, et al. Using nonlinear features for fetal heart rate classification. Biomedical Signal Processing and Control. 2012;7(4):350–357. doi: 10.1016/j.bspc.2011.06.008

[16] Henriques T, Gonçalves H, Antunes L, Matias M, Bernardes J, Costa-Santos C. Entropy and compression: Two measures of complexity. Journal of Evaluation in Clinical Practice. 2013;19(6):1101–1106. doi: 10.1111/jep.12068 23809085

[17] Costa MD, Schnettler WT, Amorim-Costa C, Bernardes J, Costa A, Goldberger AL, et al. Complexity-loss in fetal heart rate dynamics during labor as a potential biomarker of acidemia. Early Human Development. 2014;90(1):67–71. doi: 10.1016/j.earlhumdev.2013.10.002 24290526 PMC4077599

[18] Wahbah M, Al Sakaji R, Funamoto K, Krishnan A, Kimura Y, Khandoker AH. Estimating Gestational Age from Maternal-Fetal Heart Rate Coupling Parameters. IEEE Access. 2021;9:65369–65379. doi: 10.1109/ACCESS.2021.3074550

[19] Khandoker AH, Al-Angari HM, Voss A, Schulz S, Kimura Y. Quantification of maternal-fetal cardiac couplings in normal and abnormal pregnancies applying high resolution joint symbolic dynamics. Mathematical Biosciences and Engineering. 2020;17(1):802–813. doi: 10.3934/mbe.2020042 31731378

[20] Avci R, Escalona-Vargas D, Siegel ER, Lowery CL, Eswaran H. Coupling Analysis of Fetal and Maternal Heart Rates via Transfer Entropy Using Magnetocardiography. In: 2018 40th Annual International Conference of the IEEE Engineering in Medicine and Biology Society (EMBC). vol. 176. IEEE; 2018. p. 1–4. Available from: https://ieeexplore.ieee.org/document/8513053/. 10.1109/EMBC.2018.8513053 PMC8175024 30440290

[21] Pinto P, Costa-Santos C, Gonçalves H, Ayres-De-Campos D, Bernardes J. Improvements in fetal heart rate analysis by the removal of maternal-fetal heart rate ambiguities. BMC Pregnancy and Childbirth. 2015;15(1):1–7. doi: 10.1186/s12884-015-0739-1 26585345 PMC4653855

[22] Chung DY, Sim YB, Park KT, Yi SH, Shin JC, Kim SP. Spectral analysis of fetal heart rate variability as a predictor of intrapartum fetal distress. International Journal of Gynecology and Obstetrics. 2001;73(2):109–116. doi: 10.1016/S0020-7292(01)00348-4 11336729

[23] Campos I, Gonçalves H, Bernardes J, Castro L. Fetal Heart Rate Preprocessing Techniques: A Scoping Review. Bioengineering. 2024;11(4). doi: 10.3390/bioengineering11040368 38671789 PMC11048563

[24] Cowpertwait PSP, Metcalfe AV. Introductory time series with R. Springer; 2009.

[25] Castro L, Teixeira A, Brás S, Santos M, Costa-Santos C. Towards FHR Biometric Identification: A Comparison between Compression and Entropy Based Approaches. Proceedings—IEEE Symposium on Computer-Based Medical Systems. 2018;2018-June:440–441.

[26] Bras S, Pinho AJ. ECG biometric identification: A compression based approach. Proceedings of the Annual International Conference of the IEEE Engineering in Medicine and Biology Society, EMBS. 2015;2015-November:5838–5841. 10.1109/EMBC.2015.7319719 26737619

[27] Brás S, Ferreira JHT, Soares SC, Pinho AJ. Biometric and Emotion Identification: An ECG Compression Based Method. 2018;9(April):1–11. 10.3389/fpsyg.2018.00467 PMC5893853 29670564

[28] Ramos MS, Carvalho JM, Pinho AJ, Brás S. On the Impact of the Data Acquisition Protocol on ECG Biometric Identification. Sensors. 2021;21(14):4645. doi: 10.3390/s21144645 34300385 PMC8309530

[29] Chawla NV, Bowyer KW, Hall LO, Kegelmeyer WP. SMOTE: Synthetic minority over-sampling technique. Journal of Artificial Intelligence Research. 2002;16:321–357. doi: 10.1613/jair.953

[30] Sreejith S, Nehemiah HK, Kannan A. Clinical data classification using an enhanced SMOTE and chaotic evolutionary feature selection. Computers in Biology and Medicine. 2020;126(February):103991. doi: 10.1016/j.compbiomed.2020.103991 32987205

[31] Fallahi A, Jafari S. An Expert System for Detection of Breast Cancer Using Data Preprocessing and An Expert System for Detection of Breast Cancer Using Data Preprocessing and Bayesian Network. 2019;(October).

[32] Batuwita R, Palade V. microPred: effective classification of pre-miRNAs for human miRNA gene prediction. 2009;25(8):989–995. 19233894 10.1093/bioinformatics/btp107

[33] Ramya KA, Pushpa M. A Survey on Lossless and Lossy Data Compression Methods. International Journal of Computer Science and Engineering Communications. 2016;4(1):1277–1280.

[34] Gailly Jl, Adler M. Zlib compression library. 2004;.

[35] Carvalho JM, Brás S, Pinho AJ. Compression-Based Classification of ECG Using First-Order Derivatives. Lecture Notes of the Institute for Computer Sciences, Social-Informatics and Telecommunications Engineering, LNICST. 2019;273:27–36.

[36] Carvalho JM, Brás S, Pratas D, Ferreira J, Soares SC, Pinho AJ. Extended-alphabet finite-context models. Pattern Recognition Letters. 2018;112(September 2017):49–55. doi: 10.1016/j.patrec.2018.05.026

[37] Borbely RS. On normalized compression distance and large malware: Towards a useful definition of normalized compression distance for the classification of large files. Journal of Computer Virology and Hacking Techniques. 2016;12(4):235–242. doi: 10.1007/s11416-015-0260-0

[38] Costa Santos C, Vitányi PMB, Bernardes J, Antunes L. Clustering fetal heart rate tracings by compression. Proceedings—IEEE Symposium on Computer-Based Medical Systems. 2006;2006:685–690.

[39] Bagui SC. Combining Pattern Classifiers: Methods and Algorithms. vol. 47; 2005.

[40] Jin Z, Shang J, Zhu Q, Ling C, Xie W, Qiang B. RFRSF: Employee Turnover Prediction Based on Random Forests and Survival Analysis. Lecture Notes in Computer Science (including subseries Lecture Notes in Artificial Intelligence and Lecture Notes in Bioinformatics). 2020;12343 LNCS:503–515.

[41] Chen T, Guestrin C. XGBoost: A Scalable Tree Boosting System. 2016.

[42] Farquad MAH, Bose I. Preprocessing unbalanced data using support vector machine. Decision Support Systems. 2012;53(1):226–233. doi: 10.1016/j.dss.2012.01.016

[43] Devane D, Lalor JG, Daly S, McGuire W, Cuthbert A, Smith V. Cardiotocography versus intermittent auscultation of fetal heart on admission to labour ward for assessment of fetal wellbeing. Cochrane Database of Systematic Reviews. 2017;2019(5). doi: 10.1002/14651858.CD005122.pub5 28125772 PMC6464914

[44] Graham EM, Ruis KA, Hartman AL, Northington FJ, Fox HE. A systematic review of the role of intrapartum hypoxia-ischemia in the causation of neonatal encephalopathy. American Journal of Obstetrics and Gynecology. 2008;199(6):587–595. doi: 10.1016/j.ajog.2008.06.094 19084096

[45] Goorbergh RVD, Smeden MV. The harm of class imbalance corrections for risk prediction models: illustration and simulation using logistic regression. 2022;29(June):1525–1534. doi: 10.1093/jamia/ocac093 35686364 PMC9382395

